# Both Two *CtACO3* Transcripts Promoting the Accumulation of the Flavonoid Profiles in Overexpressed Transgenic Safflower

**DOI:** 10.3389/fpls.2022.833811

**Published:** 2022-04-06

**Authors:** Beixuan He, Yanjie Zhang, Lunuan Wang, Dandan Guo, Xinlei Jia, Jianhui Wu, Shuyi Qi, Hong Wu, Yue Gao, Meili Guo

**Affiliations:** ^1^Department of Pharmacognosy, College of Pharmacy, Naval Medical University (Second Military Medical University), Shanghai, China; ^2^Department of Cardiology, Changhai Hospital of Naval Medical University (Second Military Medical University), Shanghai, China

**Keywords:** safflower (C*arthamus tinctorius* L.), flavonoids biosynthesis, HSYA, regulating mechanism, *CtACO3*

## Abstract

The unique flavonoids, quinochalcones, such as hydroxysafflor yellow A (HSYA) and carthamin, in the floret of safflower showed an excellent pharmacological effect in treating cardiocerebral vascular disease, yet the regulating mechanisms governing the flavonoid biosynthesis are largely unknown. In this study, *CtACO3*, the key enzyme genes required for the ethylene signaling pathway, were found positively related to the flavonoid biosynthesis at different floret development periods in safflower and has two *CtACO3* transcripts, *CtACO3-1* and *CtACO3-2*, and the latter was a splice variant of *CtACO3* that lacked 5’ coding sequences. The functions and underlying probable mechanisms of the two transcripts have been explored. The quantitative PCR data showed that *CtACO3-1* and *CtACO3-2* were predominantly expressed in the floret and increased with floret development. Subcellular localization results indicated that *Ct*ACO3-1 was localized in the cytoplasm, whereas *Ct*ACO3-2 was localized in the cytoplasm and nucleus. Furthermore, the overexpression of *Ct*ACO3-1 or *Ct*ACO3-2 in transgenic safflower lines significantly increased the accumulation of quinochalcones and flavonols. The expression of the flavonoid pathway genes showed an upward trend, with *CtCHS1*, *CtF3H1*, *CtFLS1*, and *CtDFR1* was considerably induced in the overexpression of *CtACO3-1* or *CtACO3-2* lines. An interesting phenomenon for *Ct*ACO3-2 protein suppressing the transcription of *CtACO3-1* might be related to the nucleus location of *Ct*ACO3-2. Yeast two-hybrid (Y2H), glutathione *S*-transferase (GST) pull-down, and BiFC experiments revealed that *Ct*ACO3-2 interacted with *Ct*CSN5a. In addition, the interactions between *Ct*CSN5a and *Ct*COI1, *Ct*COI1 and *Ct*JAZ1, *Ct*JAZ1 and *Ct*bHLH3 were observed by Y2H and GST pull-down methods, respectively. The above results suggested that the *Ct*ACO3-2 promoting flavonoid accumulation might be attributed to the transcriptional activation of flavonoid biosynthesis genes by *Ct*bHLH3, whereas the *Ct*bHLH3 might be regulated through *Ct*CSN5-*Ct*COI1-*Ct*JAZ1 signal molecules. Our study provided a novel insight of *Ct*ACO3 affected the flavonoid biosynthesis in safflower.

## Introduction

Flavonoids, as a group of secondary metabolites widely existing in plants, improve the adaptation ability in the volatile and complex environment of plants (Tahara, 2007; Petroni and Tonelli, 2011). Meanwhile, the flavonoid content in medicinal plants received much attention because of its beneficial health properties against a number of diseases (Rukh et al., 2019). *Carthamus tinctorius* L., commonly known as safflower, is an important medicinal plant and widely used in treating cardiocerebral vascular disease in China. The flavonoids in safflower are the main pharmacologically active compounds, especially the unique quinochalcones, such as hydroxysafflor yellow A (HSYA) and carthamin, which have high commercial and medicinal value (Tu et al., 2015). For the deep investigation and wide application of flavonoids in safflower, it is important to explore their biosynthesis mechanism and further improve the flavonoid content in plants.

The biosynthesis pathway of basic flavonoids skeleton and the enzymes related to it have been well characterized, including phenylalanine ammonia lyase (PAL), cinnamic acid 4-hydrolase (C4H), 4-coumarate:CoA ligase (4CL), chalcone synthase (CHS), 2-hydroxyisoflavanone synthase (IFS), 2-hydroxyisoflavanone dehydratase (HID), chalcone isomerase (CHI), flavone synthase (FNS), flavanone 3-hydroxylase (F3H), flavonol synthase (FLS), flavonoid 3’-hydroxylase (F3’H), flavonoid 3’,5’-hydroxylase (F3’5’H), dihydroflavonol 4-reducatase (DFR), anthocyanidin synthase (ANS), leucoanthocyanidin 4-reductase (LAR), especially in model plant *Arabidopsis thaliana*, and a range of crop species, such as bean, tomato, maize, and rice (Tohge et al., 2017). The most of channel enzyme genes in flavonoids biosynthesis have been identified depending on transcriptome of safflower, and the function in the HSYA accumulation of *CtCHS1*, *CtCHS4*, *CtCHI1*, and *CtF3H* have been proved *in vivo* of safflower (Tu et al., 2016; Guo et al., 2017, 2019; He et al., 2018). There is broad consensus that the flavonoid pathways are regulated mostly through the coordinated transcription of structural genes by the interaction of MBW complex, such as R2R3 MYB transcription factors, basic helix–loop–helix (bHLH) transcription factors, and WD40 proteins (Payne et al., 2000; Ramsay and Glover, 2005). Some studies have reported that bHLH3, a bHLH transcription factor, played an important role in regulating the anthocyanins, flavones, and flavonols biosynthesis through the downstream channel enzyme genes (Rahim et al., 2014; Li et al., 2020; An et al., 2021).

In addition to the regulation of flavonoid metabolism channel enzyme genes and transcription factors in plants, flavonoid biosynthesis is also affected by plant hormones signaling pathway, such as methyl jasmonate (JA), auxin, and ethylene. Ethylene participates in many plants’ developmental processes and stress responses, such as plant growth, germination, flowering, fruit ripening, and senescence (Lin et al., 2009; Van de Poel et al., 2015; Wen, 2015). It is worth to mention that ethylene positivity regulates the accumulation of flavonoids implicated with various evidence. Treatment with ethylene and its precursor, 1-aminocyclopropane carboxylic acid (ACC), induced the flavonol and anthocyanin accumulation in *Arabidopsis*, apple, black carrot, and tea (Watkins et al., 2014; Barba-Espín et al., 2017; An et al., 2018; Ke et al., 2018), and isoflavone accumulation in soybean (Yuk et al., 2016). In *A. thaliana*, the anthocyanin levels were lower in the ein1-1, ein2-1, and the ein3/eil1 double mutant than in normal plants. In apple, EIN3-like1, MYB1, and ERF3 together modulate anthocyanin accumulation (An et al., 2018). Meanwhile, others reported that ethylene negatively affects anthocyanin biosynthesis. The inhibition of ethylene synthesis by aminoethoxyvinylglycine treatment increased the anthocyanin content in black rice at dark (Kumar et al., 2019). ACC acid treatment suppressed the sugar and light-inducible anthocyanin synthesis in *Arabidopsis* plants (Jeong et al., 2010). Ethylene treatment inhibited the light-induced anthocyanin and biosynthesis through the *Pp*CTR1/*Pp*ETR1 system in the red pear fruits (Ni et al., 2020). Tobacco plant carrying the mutated melon *Cm*ETR1/H69A showed higher anthocyanin level than normal plant (Keita et al., 2005). The ethylene treatment influenced the accumulation of anthocyanin in blueberry, which might depend on the cultivar (Costa et al., 2018). Accordingly, ethylene regulated the biosynthesis of flavonols and anthocyanins, in which ethylene signaling pathway has been suggested to be a regulator of anthocyanin accumulation. However, these studies have not come to an accordant conclusion, and there is little evidence demonstrating the regulatory effect of ethylene on another flavonoid biosynthesis, especially chalcones, which is the principal component in safflower.

Increasing evidence showed that ACO (ACC oxidase) has a rate-limiting role in ethylene biosynthesis, which belongs to a multigene family (Zhang et al., 2012; Houben and Van de Poel, 2019). Most ACOs were the biosynthetic structure genes of ethylene, whereas others displayed some different functions. For instance, *SlACO5* and *CsACO2*, respectively, played vital roles in low oxygen response in tomato (Sell and Hehl, 2005) and sex determination in cucumber flowers (Chen et al., 2016). In addition, the overexpression of *PtACO1* in poplar caused cambial cell division (Jonathan et al., 2009). In our previous study, the possibility of ethylene synthesis pathway regulating the accumulation of flavonoids was concerned in safflower. The overexpression of *CtACO1* reduced the accumulation of quinochalcone HSYA and carthamin, kaempferol, and its glycosylated derivatives, whereas it increased quercetin and its glycosylated derivatives (Tu et al., 2019). In the present study, it was found that the expression of *CtACO3* was closely associated with flavonoid accumulation in the floret of safflower at different development periods. The overexpression of two *CtACO3* splice variants, *CtACO3-1* and *CtACO3-2*, significantly increased the accumulation of quinochalcone HSYA and carthamin, flavonol kaempferol glycosylated derivatives, and quercetin glycosylated derivatives. An interesting phenomenon for CtACO3-2 protein suppressing the transcription of CtACO3-1 was also found. Furthermore, a possible route of *CtACO3-2* influenced flavonoid biosynthesis pathway was preliminarily explored. The following is our first report of the study.

## Materials and Methods

### Plant Materials

The safflower plant ZHH0119 (*C. tinctorius* L.), which floret with orange-yellow color and major quinochalcones, was collected from the Chinese Safflower Germplasm Resources in the Academy of Agricultural Sciences of Xinjiang. The safflower was identified by Prof. Meili Guo. It was repeatedly purified in our laboratory. The plant was grown in the greenhouse at 23 ± 2°C under the light of circadian rhythm (16-h/8- light–dark cycle) in the Naval Medical University (Shanghai, China). The voucher specimen was SMMU171201 and deposited at the Naval Medical University.

### Plasmid Construction and Safflower Transformation

The CDS of *CtACO3-1* was cloned with primers (PMT39-CtACO3-1F and CtACO3-1-PMT39R; Supplementary Table 1); *CtACO3-2* was cloned with primers (PMT39-CtACO3-2F and CtACO3-2-PMT39R; Supplementary Table 1), and empty vector PMT39 (pCAMBIA-1380-CaMV35S-MCS-EGFP-NOS) was digested by *Nco*I and made a green fluorescent protein (GFP) tag fused to the CDS of *CtACO3-1* and *CtACO3-2* and downstream of 35S promoter (Guo et al., 2017). The *Agrobacterium* strain (GV3101) including the above vector was introduced into safflower plants according to previous methods to generate overexpressing safflowers (Guo et al., 2017). Then, initial screening analyzed T1 transformants according to previous methods, the identification using primers (35SIDF, CtACO3-1IDR1, and CtACO3-2IDR1; Supplementary Table 1), the forward primer is on CaMV35S promoter, and reverse primer is on the CDS of target gene (Tu et al., 2019).

### Bioinformatics Analysis

Multiple sequence alignment was aligned in Geneious (v9.1) by the MUSCLE plugin. Then, the best-scoring maximum likelihood tree was built with 1,000 bootstrap replicates using Geneious (v9.1). Conserved protein domains were identified using SMART (Letunic et al., 2015).

### Subcellular Localization

The CDSs of *CtACO3-1* and *CtACO3-2* were cloned into the PMT-39 vector; the recombinant and control plasmids were transformed into the *Agrobacterium* strain GV3101. Positive *Agrobacterium* was cultured and cocultured with onion epidermal layers, and *N. benthamiana* leaves were injected according to previous methods (Guo et al., 2019; Tu et al., 2019). The GFP fluorescence of *Ct*ACO proteins was confirmed by a confocal microscope (Leica TCS SP5).

### RNA Extraction and Expression Analysis

The florets of *CtACO3-1*, *CtACO3-2* transgenic safflowers plants, and untreated safflower plants were collected at stage IV. Total RNA was extracted from safflower floret samples by TransZol reagents (the tubular flower without the ovary); first-strand cDNA was synthesized with the manufacturer’s instruction (TransGene Biotech, Beijing, China). Quantitative real-time PCR (qRT-PCR) was worked using TransStart Green qPCR Supermix (TransGene Biotech, Shanghai, China) with ABI7300 Real-Time PCR system (Applied Biosystems, Foster City, CA, United States). When designing Real-Time PCR primers for the transcription levels analysis of *CtACO3-1* and *CtACO3-2*, there is one pair of primers in their same region and one in the *CtACO3-1*-specific region. The relative expression level of *CtACO3-2* was confirmed by the difference between the expression level of *CtACO3-1* and *CtACO3-2* shared region and the expression level of the *CtACO3-1*-specific region. Primers used are listed in Supplementary Table 1. A quantitative reverse transcriptase–PCR thermal cycle was followed as per manufacturer’s instruction (Tm at 58°C). The results were calculated according to 2^–ΔΔCt^, whereas *Ct60s* gene (GenBank accession no. KJ634810) was used as a housekeeping gene.

### Ultra-High Performance Liquid Chromatography With Quadrupole Time-of-Flight Mass Spectrometry Detection in Safflower Samples

The preparation of above plant samples, chemicals, and reagents was followed according to previous methods (Tu et al., 2019). Agilent 6538 Accurate Mass Quadrupole Time-of-Flight MS and Agilent 1290 Infinity LC System (Agilent, Santa Clara, CA, United States) was used for Ultra-high performance liquid chromatography with quadrupole time-of-flight mass spectrometry (UHPLC-QTOF-MS) analysis. XBridge TM BEH C18 column (2.5 μm, 2.1 mm × 100 mm; Waters, Milford, MA, United States) was used for chromatographic separations. Previous methods were followed for the methods, mass spectrometer, positive ion mode, and gradient elution used for the quantification (Guo et al., 2017). The eight standard compounds were confirmed, such as D-phenylalanine (m/z 165.079), kaempferol-3-*O*-glucoside (m/z 448.1006), quercetin-3-*O*-glucoside (m/z 464.0955), rutin (m/z 610.1534), and HSYA (m/z 612.1690), purchased from Yuanye Bio-Technology (Shanghai, China), and carthamin (m/z 910.2168) was extracted in our laboratory. Agilent MassHunter quantitative analysis software was used for metabolite data.

### Yeast One-Hybrid Assay

Yeast one-hybrid (Y1H) assays were performed according to the manufacturer’s instruction of Matchmaker One-Hybrid Library Construction and Screening Kit (Clontech). The safflower cDNA library cloned in the prey vector pGAD-T7 (AD) was made by the OE BioTech. In brief, the promoter fragment of *CtACO3* (Figure 4A) was cloned into the pAbAi-bait vector, which was introduced into the yeast strain Y1H GOLD, and were cultured on SD/–Ura medium. Positive clones were sequence-verified by Matchmaker Insert Check PCR Mix 1 (Clontech), the yeast-based transcriptional activation test was followed. The screen was performed by using pAbAi-bait Y1H stain and the safflower cDNA-pGADT7-DEST library. These yeast strains were cultured on SD/–Leu medium containing 100 ng/ml AbA (Clontech). Positive clones were diluted and spotted on SD/–Leu medium containing 250 ng/ml AbA (Clontech), then sequence-verified by Matchmaker Insert Check PCR Mix 2 (Clontech).

**FIGURE 1:**
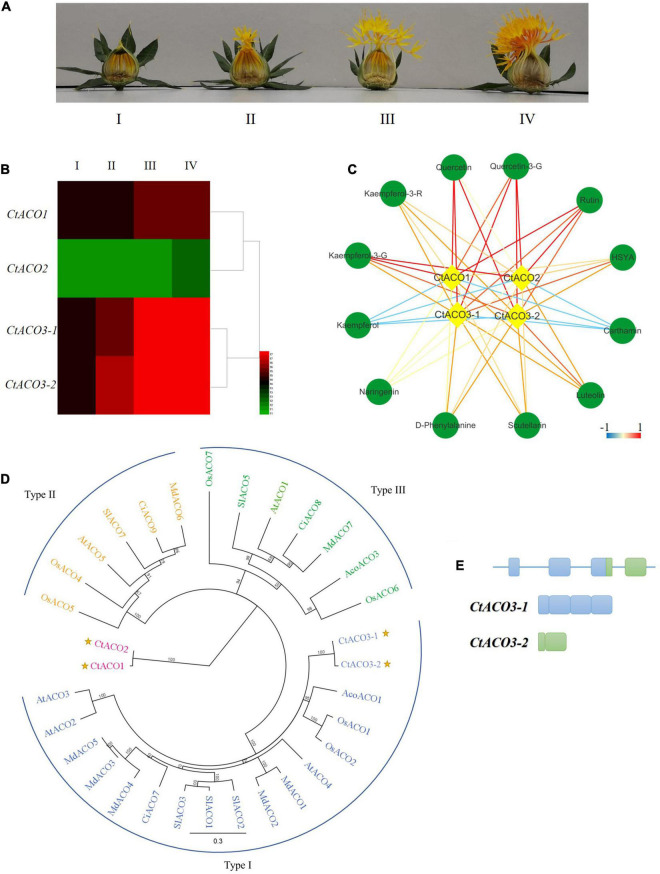
The expression pattern of ACO genes in safflower. **(A)** Different developmental stages of safflower lines. I: the day before flowering, II: the first day of flowering, III: the second to third day of flowering, IV: the full bloom. **(B)** Four *CtACOs* expression patterns at different developmental stages of safflower lines, signal values by gene chip. **(C)** The correlation coefficients between *CtACOs* and flavone glycoside compounds. The color key was set from –1 to +1. kaempferol-3-G*:* kaempferol-3-*O*-glucoside, kaempferol-3-R: kaempferol-3-rutinoside, quercetin-3-G: quercetin-3-*O*-glucoside. **(D)** Maximal likelihood phylogenetic tree for ACO protein sequences of *Carthamus tinctorius* (Ct), *Arabidopsis thaliana* (At), *Solanum lycopersicum* (Sl), *Malus domestica* (Md), *Oryza sativa* (Os), *Ananas comosus* (Aco), and *Citrus sinensis* (Ci). Type I ACO is shown in blue, Type II ACO is shown in yellow, and Type III ACO is shown in green, *Ct*ACOs are plotted. **(E)** Schematic diagram of *CtACO3* splice variants indicating introns and exons.

**FIGURE 2:**
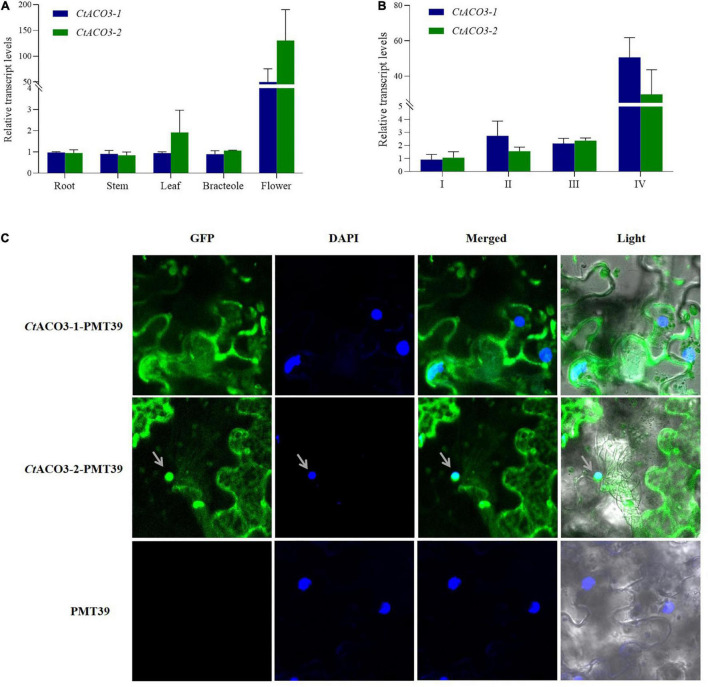
The molecular characterization of *CtACO3 splice variants*. **(A)** Relative abundance of *CtACO3-1* and *CtACO3-2* in different tissues. **(B)** Relative abundance of *CtACO3-1* and *CtACO3-2* in different floret developmental stages. **(C)** Subcellular localization of the *Ct*ACO3-1/-2-PMT39 fusion protein in *Nicotiana benthamiana* leaves. Data were expressed as means ± SD (*n* = 3).

**FIGURE 3:**
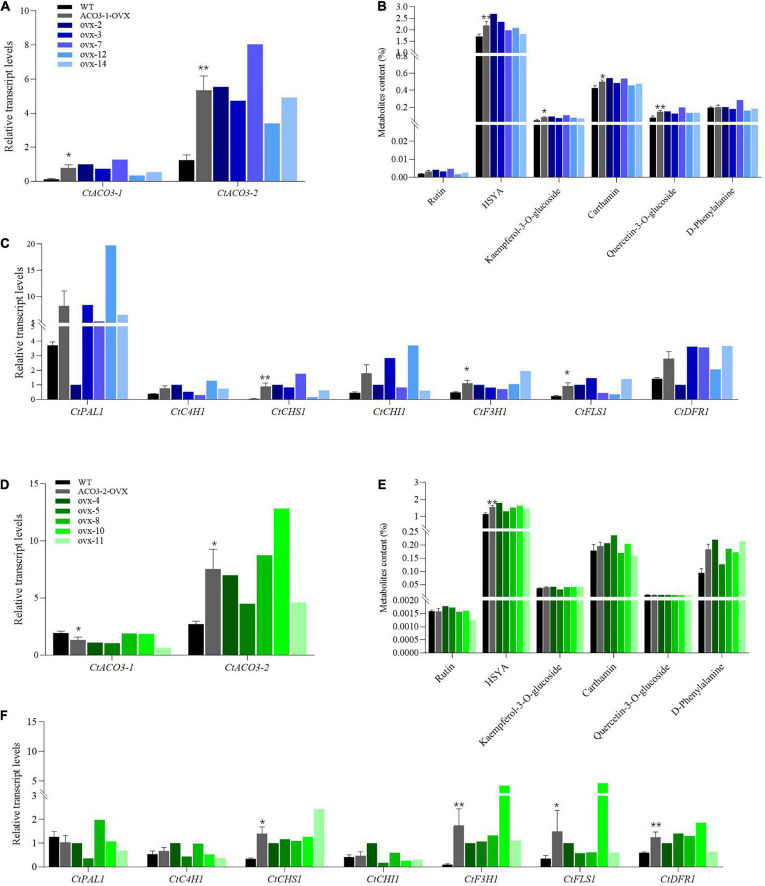
Stimulatory effect of *Ct*ACO3-1 overexpression and *Ct*ACO3-2 overexpression on main active compounds content in safflower. **(A)** Relative expression levels of *CtACO3-1* and *CtACO3-2* in WT and *Ct*ACO3-1 overexpression transgenic lines determined by quantitative PCR (qPCR). **(B)** Ultra-high performance liquid chromatography with quadrupole time-of-flight mass spectrometry (UPLC-QTOF-MS) analysis of main active compounds content in WT and *Ct*ACO3-1 overexpression transgenic lines. **(C)** The expression levels of flavonoid-related genes in WT and *Ct*ACO3-1 overexpression transgenic lines determined by qPCR. **(D)** The relative expression levels of *CtACO3-1* and *CtACO3-2* in WT and *Ct*ACO3-2 overexpression transgenic lines determined by qPCR. **(E)** UPLC-QTOF-MS analysis of main active compounds content in WT and *Ct*ACO3-2 overexpression transgenic lines. **(F)** The expression levels of flavonoid-related genes in WT and *Ct*ACO3-2 overexpression transgenic lines determined by qPCR. Data were expressed as mean ± SEM (*n* = 3), **p < 0.01, and *p < 0.05.

**FIGURE 4:**
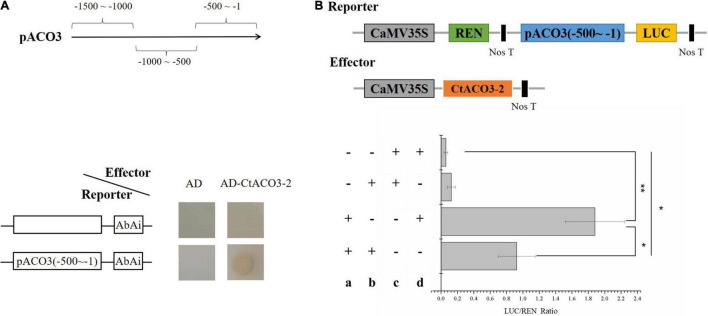
*Ct*ACO3-2 regulate the transcription of *CtACO3-1*. **(A)** Diagram of the promoter fragments of the *CtACO3* promoter. *Ct*ACO3-2 binds to the -500∼-1 fragment of *CtACO3* promoter using Y1H assay. Y1H assays were repeated for three times. **(B)** The effects of *Ct*ACO3-2 on *CtACO3* promoter deactivation. *CtACO3* promoter p*Ct*ACO3 (-500∼-1) was fused to LUC reporter and the promoter activity was determined by a transient Dual-LUC assay in *N. benthamiana.* The relative LUC activity was normalized to the reference Renilla (REN) luciferase. (a): Reporter, (b): effector, c: pGreenII0800-LUC, d: pGreenII 62-SK. Data were expressed as mean ± SD (*n* = 3), **p < 0.01, and *p < 0.05.

### Dual-Luciferase Reporter Assay

To confirm the interaction between *Ct*ACO3-2 and the promoter of *CtACO3*, the CDS of *CtACO3-2* was inserted into pGreenII 62-SK, and the promoter pACO3 (-500 to -1) of *CtACO3* was cloned into pGreen 0800-LUC.

The constructed effector pGreenII 62-SK-CtACO3-2, reporter plasmids pACO3-LUC and control vector pGreenII 62-SK, and pGreen 0800-LUC were introduced into *Agrobacterium* strain GV3101 (pSoup-19T), respectively. Mixed bacteria solution harbored the effector and reporter (1:1), which was injected into tobacco leaves. After 4 days, a dual-luciferase assay kit was used to measure LUC and REN luciferase activities (Promega) following Liu et al. (2013) Three biological repeats were assayed for each combination. The results were calculated using the ratio of LUC to REN.

### Yeast Two-Hybrid Assay

Yeast two-hybrid (Y2H) screening and Y2H assays were performed following the manufacturer’s instructions (Clontech, Mountain View, CA, United States). The CDS of *CtACO3-2*, *CtCSN5a*, *CtCOI1*, *CtJAZ1*, and *CtbHLH3*; the C-terminal of *CtCOI1* and *CtbHLH3*; and the N-terminal of *CtCOI1* and *CtbHLH3* were inserted into the pGBKT7 or pGADT7 vector to fuse with the DNA-BD and AD, respectively (primers are listed in Supplementary Table 1). Autoactivation and suppression of autoactivation of the bait constructs were tested by cultured in SD/-Trp medium 40 mg/ml X-α-Gal. Then, bait constructs without self-activation were transformed into Y2H GOLD strain with prey constructs using the lithium acetate method and cultured in DDO (SD/-Leu/-Trp) medium containing 125 ng/ml AbA and 40 mg/ml X-α-Gal for 5 days. Then, transformed positive colonies were plated onto QDO (SD/-Ade/-His/-Leu/-Trp) medium containing 125 ng/ml AbA and 40 mg/mL X-α-Gal, and positive clones were verified by using a Matchmaker Insert Check PCR Mix 2 (Clontech).

### Glutathione *S*-Transferase Pull-Down Assay

For the construction of GST-*Ct*CSN5a, GST-*Ct*COI1, GST-*Ct*bHLH3N, His-*Ct*ACO3-2, and His-*Ct*JAZ1 expression vectors, the CDS of *CtCSN5a*, *CtACO3-2*, *CtCOI1*, *CtJAZ1*, and *CtbHLH3N* was cloned into pGEX-6P-1 or pET-32a, respectively. To test whether *Ct*ACO3-2 interacts with *Ct*CSN5a protein, *Ct*CSN5a interacts with *Ct*COI1, *Ct*COI1 interacts with *Ct*JAZ1, and *Ct*JAZ1 interacts with *Ct*bHLH3N, according to the manufacturer’s instruction for the Pierce GST Protein Interaction Pull-Down Kit (Thermo Scientific). Briefly, His-bait fusion protein was incubated with GST-prey fusion proteins with slowly shaking for a night. Then, beads were washed five times and heated for 5 min in 100°C. Sodium dodecyl sulfate–polyacrylamide gel electrophoresis (SDS-PAGE) and Western blotting were used to confirm the proteins by anti-GST (Beyotime, 1/1,000) and anti-His (Beyotime, 1/1,000) antibodies, respectively.

### Bimolecular Fluorescence Complementation Analysis

The CDSs of *CtACO3-1 and CtACO3-2* were inserted in the pCAMBIA1300-35S-NY173 vector to create *Ct*ACO3-1-nYFP and *Ct*ACO3-2-nYFP constructs. Similarly, the CDSs of *CtCSN5a* were inserted in the pCAMBIA1300-35S-YC155 vector. The specific primers used for *Ct*ACO3-1-nYFP, *Ct*ACO3-2-nYFP, and *Ct*CSN5a-cYFP construction are described in Supplementary Table 1. Then, the construction was introduced into *Agrobacterium* GV3101 strain subsequently. The mixed bacteria solution containing nYFP and cYFP pairs was injected into tobacco leaves with a syringe and grown for 4 days. The confocal microscope was used for YFP fluorescence detection (Leica TCS SP5).

### Statistics

The data are presented as mean ± standard deviation (SD) or mean ± standard error of mean (SEM) and analyzed using GraphPad Prism 8 software (GraphPad Software, La Jolla, CA, United States). A paired two-tailed Student’s *t*-test was used to compare group differences. The value of *p* < 0.05 was regarded as statistically significant.

## Results

### Expression of *Ct*ACOs Was Related to the Accumulation of Safflower Flavonoids

In our previous study, a normalized cDNA library and gene chip data of safflower were analyzed systematically (Guo et al., 2017). Three genes were annotated as ACO enzymes in safflower line, there were two *CtACO3* splice variants, *CtACO3-1* and *CtACO3-2.* We analyzed the transcription levels of *CtACOs* and the contents of main flavonoids at different flowering times (Figures 1A,B). The coexpression analysis of “gene metabolites” is displayed in Figure 1C. Results indicate that *CtACO* family genes are positively related to most of flavonoid in safflower lines.

### *CtACOs* Phylogeny and Residue Analysis

There were a few reports that classified three distinct phylogenetic groups in the ACOs (Jafari et al., 2012). *Ct*ACOs and some homologous proteins from other plants were analyzed by phylogenetic tree analysis, showing three clusters of ACOs (Houben and Van de Poel, 2019). *Ct*ACO3 within the type I ACO cluster which exhibits high sequence similarity with *Aco*ACO1, *Os*ACO1, and *Os*ACO2, but *Ct*ACO1 and *Ct*ACO2 are not within any ACO clusters of these three types (Figure 1D). Furthermore, a detailed residue analysis of these ACO alignments is presented in Supplementary Figure 1. It has been reported that the ACO types can be classified by the intermediate residue presented in the conserved RXS motif, such as type I (R-M-S), type II (R-L/I-S), and type III (R-R-S) (Houben and Van de Poel, 2019). Interestingly, *Ct*ACO1 and *Ct*ACO2 consist of R-V-S, which was different from those three types. In parallel, all of *Ct*ACOs have conserved 2-His-1-carboxylate Fe (II) binding motif. Residues Q273, K284, K321, and F400 are conserved in *Ct*ACOs, which are important for ACO activity according to DR (Dilley et al., 2013). This could partially account for the different roles of *Ct*ACOs on flavonoid accumulation in safflower.

### Molecular Characterization of *CtACO3* Splice Variants

The *CtACO3-1* (GenBank accession no. MH67444) is a full-length transcript corresponding to the coding sequence of *CtACO3* and was predicted to encode a protein of 345 amino acids, with a molecular mass of 36.16 kD and a calculated pI of 6.57. The *CtACO3-2* transcript (GenBank accession no. MW075467) encodes a truncated protein of 110 amino acid, with a molecular mass of 12.48 kD and a calculated isoelectric point (pI) of 7.40, in which the start codon is located at the 4th exon, lacking the 235 N-terminal residues (Figure 1E). Conserved domain analyses indicate that in the C-terminal regions, *Ct*ACOs contain a conserved C3HC4 RING finger domain (Supplementary Figure 1).

To detect the expression patterns of *CtACO3-1* and *CtACO3-2* at different flowering times (I, II, III, and IV) and specific tissues (flower, leaf, bracteole, and stem), the plant materials were collected. The transcript levels of *CtACO3-1* and *CtACO3-2* in flowers increased continuously with the floret flowering, shown in Figure 2A. Both *CtACO3-1* and *CtACO3-2* showed the highest transcript level in flower (Figure 2B), whereas CtACO1 had the highest level in leaf (Tu et al., 2019).

The *CtACO3-1* and *CtACO3-2* were coexpressed with GFP in onion epidermal cells and *Nicotiana benthamiana* leaves to identify the subcellular localization. The result indicates that *CtACO3-1* localized to the cytoplasm, and *CtACO3-2* localized to the cytoplasm and nucleus (Figure 2C and Supplementary Figure 2), which were different from the cytosol location of *Ct*ACO1.

### Profiling of Flavonoid Accumulation in *CtACO3-1*-Overexpression Safflower and *CtACO3-2*-Overexpression Safflower

To explore the function of *CtACO3-1* and *CtACO3-2 in vivo* of safflower, transgenic safflower plants that overexpressed *CtACO3-1* and *CtACO3-2* under cauliflower mosaic virus (CaMV) 35S promoter were generated. In total, 10 independent positive *CtACO3-1*-overexpression transgenic lines and eight independent positive *CtACO3-2*-overexpression transgenic lines were screened out by genomic DNA PCR (Supplementary Figures 3, 4) and compared with untreated safflower lines (wild type); the relative transcription level of *CtACO3-1* increased significantly in *CtACO3-1*-overexpression plants and had the highest expression level in ovx7 (∼10.5-fold), whereas a higher level was found in ovx2 (∼8.3-fold) and ovx3 (∼6.12-fold) lines (Figure 3A); the relative transcription level of *CtACO3-2* showed the highest expression level in ovx10 (∼4.7-fold), whereas a higher level was found in ovx8 (∼3.2-fold) and ovx4 (∼2.5-fold) lines (Figure 3D). As shown in Supplementary Figure 5, there was almost no difference in plant appearance and growth status between the untreated and the transgenic plants. Five *CtACO3-1*-overexpression lines (nos. 2, 3, 4, 12, 14) and five *CtACO3-2*-overexpression lines (nos. 4, 5, 8, 10, and 11) were used to further analyze the profiling of flavonoids in safflower. The levels of flavonoid metabolites were measured by UPLC–electrospray ionization–QTOF-MS. It is shown that most of flavonoid accumulation enhanced in *CtACO3-1*-overexpression safflower lines and *CtACO3-2*-overexpression ones, especially the four mage compounds, quinochalcones (HSYA and carthamin), and flavonols (quercetin-3-*O*-glucoside and kaempferol-3-*O*-glucoside). In *CtACO3-1*-overexpression safflower lines, HSYA increased 56.79, 36.77, and 21.45% in ovx-2, ovx-3, and ovx-12 lines, respectively. Moreover, carthamin increased most robustly in the ovx-2 line (∼27.29%) and second most robustly in the ovx-7 line (∼26.21%). Quercetin-3-*O*-glucoside and kaempferol-3-*O*-glucoside were increased in each overexpression *CtACO3* safflower plant (50–160%) (Figure 3B). Besides, in *CtACO3-2*-overexpression safflower lines, HSYA increased 59.34, 43.67, and 35.69% in ovx-4, ovx-10, and ovx-8 lines, respectively. Moreover, quercetin-3-*O*-glucoside increased most robustly in the ovx-11 line (∼600%) and second most robustly in the ovx-10 line (∼500%), whereas carthamin was slightly increased in each overexpression *CtACO3-2* overexpression safflower plant (Figure 3E). In brief, the overexpression of *CtACO3-1* and *CtACO3-2* in safflower resulted in the most increase of flavonoids in flowers, and the metabolic flux of the flavonoid pathway was suggested to be directed into both the quinochacone and flavonol branch.

### Transcriptional Expression of Associated Genes in *CtACO3-1*-Overexpression Safflower and *CtACO3-2*-Overexpression Safflower

To further explore the flavonoid biosynthesis in *CtACO3-1*-overexpression safflower and *CtACO3-2*-overexpression safflower, the transgenic plants were used to investigate the transcript abundance of flavonoid biosynthesis–related genes, such as *CtPAL1*, *CtC4H1*, *CtCHS1*, *CtCHI1*, *CtF3H1*, *CtFLS1*, and *CtDFR1*, which displayed different expression pattern in CtACO3-1-overexpression safflower lines and *CtACO3-2*-overexpression safflower lines. As shown in Figure 3C, the transcript levels of upstream genes of the flavonoid pathway, such as *CtPAL1*, *CtC4H1*, and *CtCHI1*, shown an upward trend with *CtCHS1* significantly increased in the *CtACO3-1-*overexpression lines. The expression of downstream genes *CtF3H1* and *CtFLS1* was considerably induced. Similarly, the transcript levels of *CtC4H1* and *CtCHI1* in *CtACO3-2*-overexpression safflower lines shown an upward trend as well, the expression of *CtCHS1*, *CtF3H1*, *CtFLS1*, and *CtDFR1* was induced (Figure 3F). Overall, the transcript abundance of flavonoid biosynthesis–related genes performed similar trends after *CtACO3-1* or *CtACO3-2* overexpression. An additional interesting phenomenon was unraveled when we analyzed the transcript levels of other *CtACOs* in *CtACO3-1* or *CtACO3-2*-overexpression safflower. Interestingly, *CtACO3-1*-overexpression safflowers had a higher transcript level of *CtACO3-2*, whereas the level of *CtACO3-1* was lower in *CtACO3-2*-overexpression safflowers. These results indicate a feedback regulation between *Ct*ACO3-2 and *CtACO3-1*, and *CtACO3-2* may be the reason that *CtACO3-2* and *CtACO3-1* overexpression resulted in different flavonoid accumulation.

### *Ct*ACO3-2 Regulates the Transcription of *CtACO3-1 in vitro* and *in vivo*

The promoter of *CtACO3-1* contained many *cis*-elements (Supplementary Figure 6), such as G-box (5′-CACGTG-3′). To determine whether the molecules related to flavonoid biosynthesis directly regulate the transcription of *CtACO3-1*, Y1H assays were conducted. The promoters of *CtACO3* were divided into three fragments, namely, pCtACO3 (-1,500 to -1,000), pCtACO3 (-1,000 to -500), and pCtACO3 (-500 to -1), and fused to the pAbAi vector, respectively; only promoter pCtACO3 (-500 to -1) exhibited no transcriptional activation activity in the yeast-based transcriptional activation test. Moreover, the results showed that *Ct*ACO3-2 could specifically bind to the promoter pCtACO3 (-500 to -1) of *CtACO3-1* (Figure 4A).

To further clarify the regulatory effect of *Ct*ACO3-2 on *CtACO3-1* transcription, pCtACO3 (-500 to -1) was fused to the LUC to generate reporter constructs pCtACO3:LUC. Meanwhile, *Ct*ACO3-2 driven by CaMV 35S promoter was used as an effector construct. The pairs of effector and reporter were coexpressed in tobacco. When there was a presence of *Ct*ACO3-2 protein in the infiltration mixture, the luciferase (LUC)/Renilla (REN) values were significantly decreased by 49% for pCtACO3 (-500 to -1), compared with the control (Figure 4B). This resulted from the dual-luciferase assays that suggested *Ct*ACO3-2 downregulated *CtACO3-1* at the transcriptional level.

### *Ct*ACO3-2 Interacts With the COP9 Signalosome Subunit 5

**FIGURE 5:**
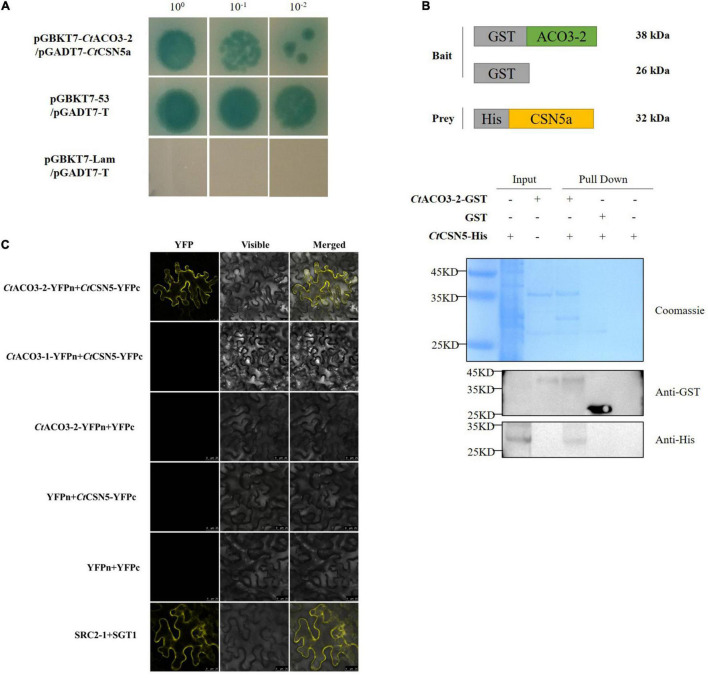
*Ct*ACO3-2 interacts with the COP9 signalosome (CSN) subunit 5. **(A)** Yeast two-hybrid (Y2H) assay showing that *Ct*ACO3-2 interact with *Ct*CSN5a. **(B)** Glutathione S-transferase (GST)-pull down assay showing that *Ct*CSN5 associates with *Ct*ACO3-2 *in vitro*. Purified GST-*Ct*ACO3-2 or GST proteins were bound to glutathione-sepharose beads, and then incubated with His-*Ct*CSN5a. Here, we show the protein bands of resulting pull-down products after western blotting with anti-GST and anti-His antibodies. **(C)** Bifc assay showing that *Ct*CSN5 interact with *Ct*ACO3-2 but not *Ct*ACO3-1 *in vivo*.

To understand how *Ct*ACO3-1 and *Ct*ACO3-2 participate in affecting the flavonoid accumulation in safflower, we used the Y2H system to identify its potential interaction partners. The CDS of *Ct*ACO3-2 constructed a bait vector [*Ct*ACO3-2-binding domain (BD)]. The bait and a library of cDNAs containing inserts for prey proteins fused to GAL4–activation domain (AD) were cotransformed to Y2H GOLD. After screening, 12 independent clones were identified, and the information is shown in Supplementary Table 2 and Supplementary Figure 7. To confirm the interaction of the clones about flavonoid biosynthesis with *Ct*ACO3-2 in yeast, the CDS of *Ct*CSN5a was fused to AD vector and cotransformed into Y2H GOLD with *Ct*ACO3-2-BD; the interactions were reconstructed (Figure 5A).

Our research then demonstrated that *Ct*ACO3-2 was associated with *Ct*CSN5a (GenBank accession no. MW075465) using pull-down assay *in vitro* and BiFC assay *in vivo* (Figures 5B,C). Therefore, these results strongly indicate that *Ct*ACO3-2 is physically associated with *Ct*CSN5a *in vitro* and in plant.

### CSN Subunit 5 Interacts With *Ct*COI1 and *Ct*COI1 Regulating the Flavonoid Accumulation Through *Ct*JAZ1 and *Ct*bHLH3 *in vitro*

**FIGURE 6:**
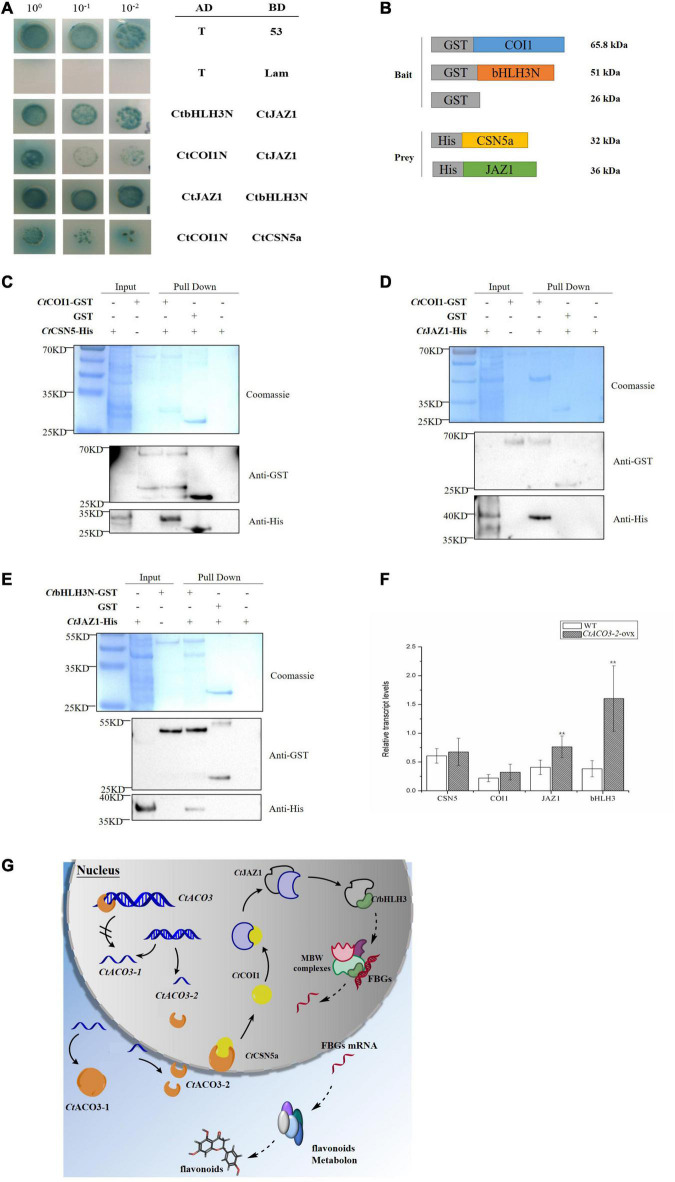
Preliminary molecular mechanism of *Ct*ACO3-2 regulates the biosynthesis pathway of flavonoids. **(A)** Yeast two-hybrid assay showing the protein–protein interaction of *Ct*CSN5-*Ct*COI1-*Ct*JAZ1-*Ct*bHLH3. **(B–E)** Demonstration of *Ct*CSN5-*Ct*COI1-*Ct*JAZ1-*Ct*bHLH3 interaction by GST pull-down assay. Purified GST-*Ct*COI1, GST-*Ct*bHLH3N, or GST proteins were bound to glutathione-sepharose beads, and then incubated with His-*Ct*CSN5a or His-*Ct*JAZ1. Here, we show the protein bands of resulting pull-down products after western blotting with anti-GST and anti-His antibodies. **(F)** The relative transcript levels of *Ct*CSN5a, *Ct*COI1, *Ct*JAZ1, and *Ct*bHLH3 in *CtACO3-2* overexpression lines. Data were expressed as mean ± SD (*n* = 8), **p < 0.01. **(G)** Schematic diagram illustrating the preliminary molecular mechanism of *Ct*ACO3-2 regulate the biosynthesis pathway of flavonoids. The solid line represents the interactions have been proved in the study, the dotted line represents the interactions have not been proved. Arrowheads indicate activations and “//” on arrow indicate inhibitions. The left of figure is two transcripts of *CtACO3*, and *Ct*ACO3-2 inhibits the transcription of *CtACO3-1*; the right of figure is the network that *Ct*ACO3-2 influence the accumulation of flavonoids through *Ct*CSN5-*Ct*COI1-*Ct*JAZ1-*Ct*bHLH3.

Wei et al. (2018) have reported that CSN subunit 5 enhanced *MYB75* and suppressed *GL2* and other genes associated with the TTG1/basic helix–loop–helix (bHLH)/MYB complexes to regulate anthocyanin accumulation. To understand how *Ct*CSN5a participated in flavonoid accumulation regulation in safflower, Y2H screening assay was used to search the proteins that interact with *Ct*CSN5a-BD. After screening and confirming, *Ct*COI1 (GenBank accession no. MW075466) interacted with *Ct*CSN5a (Supplementary Table 3). COI1 as a subunit of SCF (COI1) E3 ubiquitin ligase encodes an F-box protein, which is required for JA responses. It has been reported that COI1 interacted directly with CSN (Feng et al., 2003). Therefore, we speculated that *Ct*CSN5a regulated the accumulation of flavonoids in safflower through interaction with *Ct*COI1 (Figures 6A–C).

Jasmonate ZIM-domain (JAZ) protein family as a key regulator of JA signaling has been reported physically interacting with SCF^COI1^ (Thines et al., 2007). There is broad consensus that the flavonoid pathways are regulated mostly through the coordinated transcription of structural genes by the interaction of MBW complex, such as R2R3 MYB transcription factors, bHLH transcription factors, and WD40 proteins (Payne et al., 2000; Ramsay and Glover, 2005). An et al. reported that JAZ protein interacts with *Md*bHLH3, which belongs to MBW complex, to regulate the accumulation of anthocyanins in apple (Ni et al., 2020). In the present study, the overexpression of *CtACO3-2* resulted in the higher level of downstream structural genes. It is consistent with previous studies in which bHLH3 increased the transcription of downstream structural genes in mulberry fruits and apples (Li et al., 2020; An et al., 2021). The results therefore provided strong evidence for the hypothesis. To further test the hypothesis that JAZ1 interacts with SCF^COI1^ and bHLH3 in safflower, we examined a possible physical interaction between *Ct*COI1 and *Ct*JAZ1, *Ct*bHLH3, and *Ct*JAZ1 using the Y2H system, respectively (Figure 6A). To determine whether JAZ proteins interact with COI1 or bHLH3 *in vitro*, *Ct*COI1-GST and *Ct*bHLH3N-GST were performed with *Ct*JAZ1-HIS (Figures 6D,E). Taken together, it was demonstrated that *Ct*JAZ1 (GenBank accession no. MW075468) could physically interact with *Ct*COI1 and *Ct*bHLH3 (GenBank accession no. MW075469), respectively. It should be noted that these interactions have been examined only *in vitro*, and the preliminary model is novel and large (Figures 6F,G). The present study may provide novel ideas for flavonoids regulatory network in safflower, but further research is still required.

## Discussion

As a representative bulk Chinese medicine product, a growing number of research about safflower have been demonstrated from a molecular point of view. Results presented in this study revealed the diverse molecular characteristics of *Ct*ACO3-2, which influenced the flavonoid accumulation in safflower.

In safflower, the transcript levels of *CtACOs* in flower were all increased continuously with the floret flowering. However, the expression of different *CtACOs* showed tissue specificity; *CtACO1* had the highest expression in leaf (Tu et al., 2019), whereas both *CtACO3-1* and *CtACO3-2* expression peaked in flower. These results were in line with the previous research, that ACO had multiple expression characteristics temporally and spatially (Barry et al., 1996; Nakatsuka et al., 1998; Brady et al., 2007; Park et al., 2018). *Ct*ACO3-1 is localized in cytosol, whereas *Ct*ACO3-2 is localized not only in cytosol but also in the nucleus. The different residues of *Ct*ACOs may be the reason for their different characteristics.

Only a few transcriptional factors have been identified for regulating ACO expression (Houben and Van de Poel, 2019), such as *Sl*HB-1 in tomato (Lin et al., 2008), *Ma*ERF11 in banana (Han et al., 2016), *Cm*EIN3-like in melon fruit (Huang et al., 2010), and *Cs*WIP1in cucumber (Chen et al., 2016). In the present study, *Ct*ACO3-2 banded to the promoter of *CtACO3* and repressed the transcription of *CtACO3-1*. *Ct*ACO3-2 is a splice variant of *Ct*ACO3 and lacked 5′ coding sequences, which might be the similar manner of TOC1, an autoregulatory response regulator, in *Arabidopsis* (Strayer et al., 2000). TOC1 encodes a nuclear protein and participates in a feedback loop to control its own expression, and *Ct*ACO3-2 encodes a nuclear protein to control *Ct*ACO3-1, the full-length transcript expression (Figure 6G). In present study, *CtACO3-1*-overexpression safflowers had a higher transcript level of *CtACO3-2*, whereas the level of *CtACO3-1* was lower in *CtACO3-2*-overexpression safflowers. The above phenomenon may be because of the transcriptional regulation of *CtACO3-2*, while there is not sufficient evidence regarding the transcriptional factor function of *Ct*ACO3-2, but this finding deserves further exploration. In parallel, we do not discount the possibility of that the transcription level of *Ct*ACO3-1 and *Ct*ACO3-2 were coordinated by a transcriptional network as ACOs.

The overexpression of *CtACO3-1* and *CtACO3-2* promote the accumulation of quinochalcone and flavonol glycosylated derivatives, such as HSYA, carthamin, quercetin glycosylated derivatives, and kaempferol glycosylated derivatives in the present study, whereas *Ct*ACO1 suppressed the flavonoid accumulation (Tu et al., 2019). There have been numerous reports on the synthesis of flavonoids regulated by ethylene, *Ct*ACO as the key enzyme genes required for ethylene signaling pathway, which may further affect the flavonoid biosynthesis by regulating ethylene synthesis. Meanwhile, we preliminarily constructed a novel pathway, that *Ct*ACO3-2 regulated the biosynthesis of flavonoids by *Ct*CSN5a. That may be the reason why *Ct*ACOs play various roles in flavonoid accumulation particularly in HSYA biosynthesis, which may be helpful in further work on studying the functions of ACOs, as well as regulating the metabolic flux of active compounds in safflower by appropriate genetic engineering strategies.

At the last step of the ethylene biosynthesis, ACO interacted with biomolecules mostly about that. For example, the flower senescence was affected by the interaction between ACO1 and GRL2 in petunia (Tan et al., 2014). In this study, we identified that *Ct*EXLB (expansion-like) interacted with *Ct*ACO3-2 (Supplementary Table 2 and Supplementary Figure 7), as an effective factor of cell division participating in plant development and senescence. At the same time, CSN subunit 5 was found to interact with *Ct*ACO3-2 affecting flavonoid accumulation in safflower. Dohmann et al. (2005) reported that the CSN subunit 5 could enhance anthocyanin production in the loss-function *Arabidopsis* mutants (Dohmann et al., 2005), and CSN subunit 5 could also enhance MYB75 and suppress GL2 expressions associated with the MBW complexes through anthocyanin accumulation regulation (Wei et al., 2018). From the Y2H and GST pull-down results, it is indicated that *Ct*CSN5a interacted with *Ct*COI1 protein, mediated the interaction between JAZ1 and bHLH3, directly bound to the promoter of flavonoid biosynthesis structural genes, and regulated their transcription. The protein interaction data in the current study provide *Ct*CSN5a protein as the bridge of *Ct*ACO3-2 and *Ct*COI1 protein, whereas *Ct*ACO3-2 belongs to the ethylene biosynthesis pathway, and *Ct*COI1 belongs to the JA signaling pathway.

**FIGURE 7:**
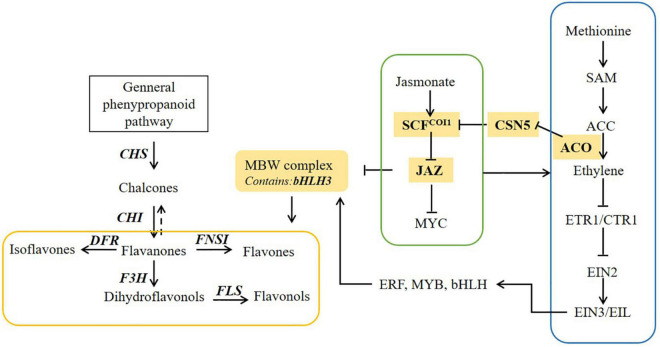
The proposed pathway underlying ethylene and jasmonate (JA) pathway induced flavonoid biosynthesis in safflower.

Flavonoid biosynthesis is regulated by diverse plant hormones, such as ethylene and JA (Flores and Ruiz del Castillo, 2014; An et al., 2018). Rudell and Mattheis reported that JA and ethylene could induce anthocyanin accumulation in apple fruits, synergistically (Rudell and Mattheis, 2008). Ni et al. (2020) found that ethylene could mediate the branching of the JA-induced flavonoid biosynthesis pathway in the red Chinese pear fruit. In safflower, exogenous application of methyl JA increases the accumulation of mostly flavonoids in safflower shown in our previous study (He et al., 2018). This study offers new insight for the common effects of ethylene and JA on flavonoid accumulation in safflower (Figure 7). The accumulation of active ingredients in botanicals is regulated by extensive networks, such as salvianolic acid in *Salvia miltiorrhiza*, Artemisinin in *Artemisia annua* (Lv et al., 2017; Deng et al., 2020a,b; Fu et al., 2020; Hao et al., 2020). Therefore, there is a long way to study the acting factors and regulatory networks of flavonoids biosynthesis in safflower.

This study is limited by that the genetic background remains unclear, the medicinal ingredients accumulate in flower and the tissue culture system still difficult. So, in the present study, we were unable to achieve the knockout or knockdown to further verify the function of gene from the opposite side. Besides, the principal limitations of the present study were that the validation of the regulatory network that *Ct*ACO3-2 regulates flavonoid synthesis *via Ct*CSN5a was performed only *in vitro* experiments, further research *in vivo* is required.

## Data Availability Statement

The datasets presented in this study can be found in online repositories. The names of the repository/repositories and accession number(s) can be found in the article/Supplementary Material.

## Author Contributions

BH, YZ, and LW contributed to most of the experiments, data analysis, and writing of the manuscript. BH, DG, XJ, JW, and SQ contributed with reagents, materials, and assisted in doing the experiment. MG, YG, and HW designed the experiments, suggested the manuscript outline and guided the writing of the manuscript, and data analysis. All authors read and approved the final manuscript.

## Conflict of Interest

The authors declare that the research was conducted in the absence of any commercial or financial relationships that could be construed as a potential conflict of interest.

## Publisher’s Note

All claims expressed in this article are solely those of the authors and do not necessarily represent those of their affiliated organizations, or those of the publisher, the editors and the reviewers. Any product that may be evaluated in this article, or claim that may be made by its manufacturer, is not guaranteed or endorsed by the publisher.

## Supplementary Material

Supplementary Figure 1 Sequence alignment for ACO protein sequences of *Carthamus tinctorius* (Ct), *Arabidopsis thaliana* (At), *Solanum lycopersicum* (Sl), *Malus domestica* (Md), *Oryza sativa* (Os), *Ananas comosus* (Aco), and *Citrus sinensis* (Ci). Type I ACO is shown in blue, Type II ACO is shown in yellow, and Type III ACO is shown in green, CtACOs are boxed.

Supplementary Figure 2 Subcellular localization of the *Ct*ACO3-PMT39 fusion protein in onion epidermal cells.

Supplementary Figure 3 Representative PCR analyses for the specific genes of *CtACO3-1*-overexpressing plants.

Supplementary Figure 4 Representative PCR analyses for the specific genes of *CtACO3-2*-overexpressing plants.

Supplementary Figure 5 Phenotypic difference between WT and *CtACO*-overexpressing plants.

Supplementary Figure 6 Cis-elements in the promoter region of *CtACO3*-1.

Supplementary Figure 7 Proteins interact with *Ct*ACO3-2 by Y2H.

Supplementary Table 1 PCR primers used in the text.

Supplementary Table 2 Proteins interact with *CtACO3*-2 by Y2H.

Supplementary Table 3 Proteins interact with *CtCSN5a* by Y2H.
