# Supplementary figures and images for: Both Two CtACO3 Transcripts Promoting the Accumulation of the Flavonoid Profiles in Overexpressed Transgenic Safflower
Source: Front Plant Sci. 2022 Apr 6;13:833811. doi: 10.3389/fpls.2022.833811 (PMC9019494; doi:10.3389/fpls.2022.833811)

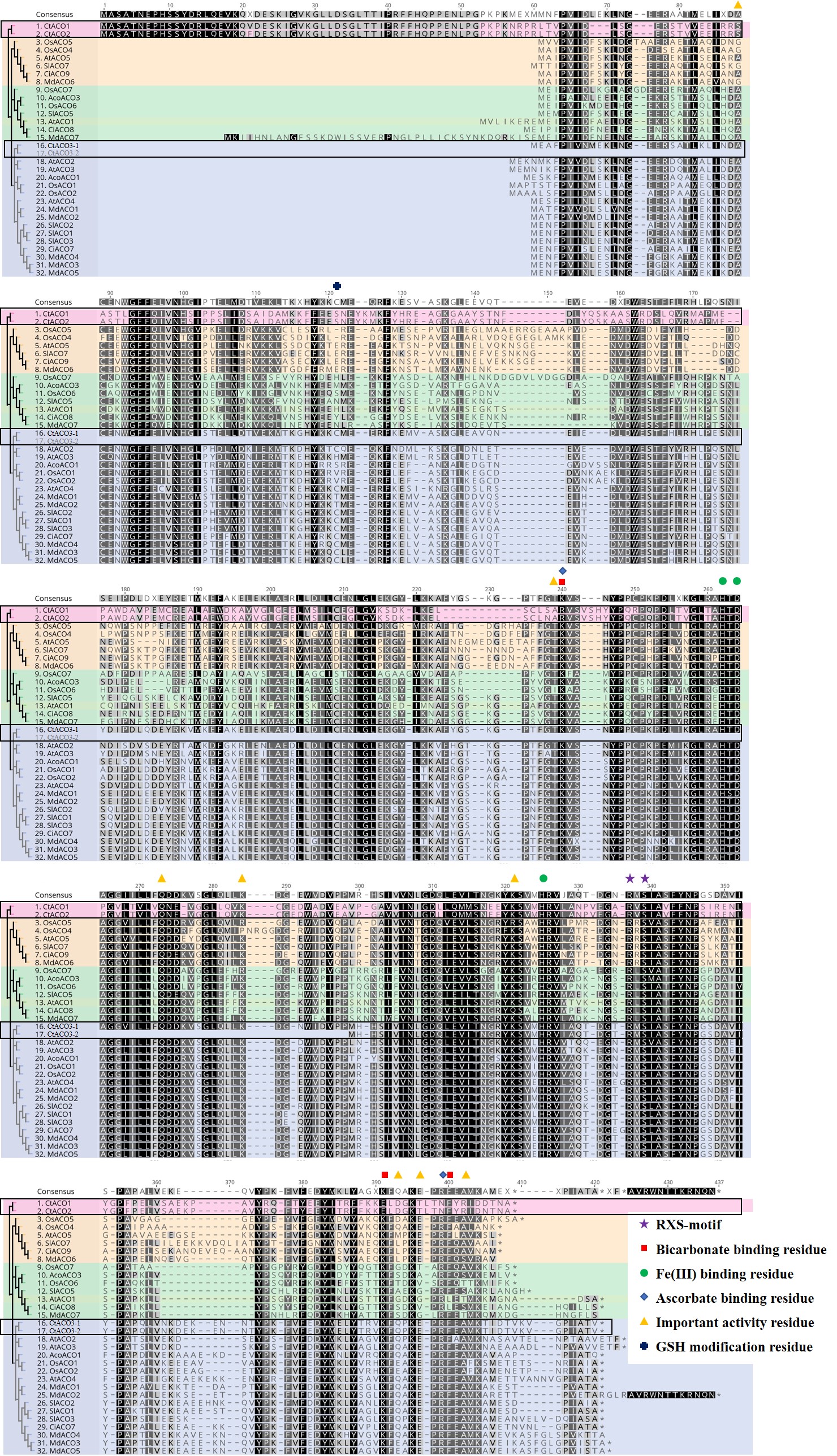

Supplement: Supplementary Figure 1 — Sequence alignment for ACO protein sequences of Carthamus tinctorius (Ct), Arabidopsis thaliana (At), Solanum lycopersicum (Sl), Malus domestica (Md), Oryza sativa (Os), Ananas comosus (Aco), and Citrus sinensis (Ci). Type I ACO is shown in blue, Type II ACO is shown in yellow, and Type III ACO is shown in green, CtACOs are boxed. [file Image_1.JPEG]

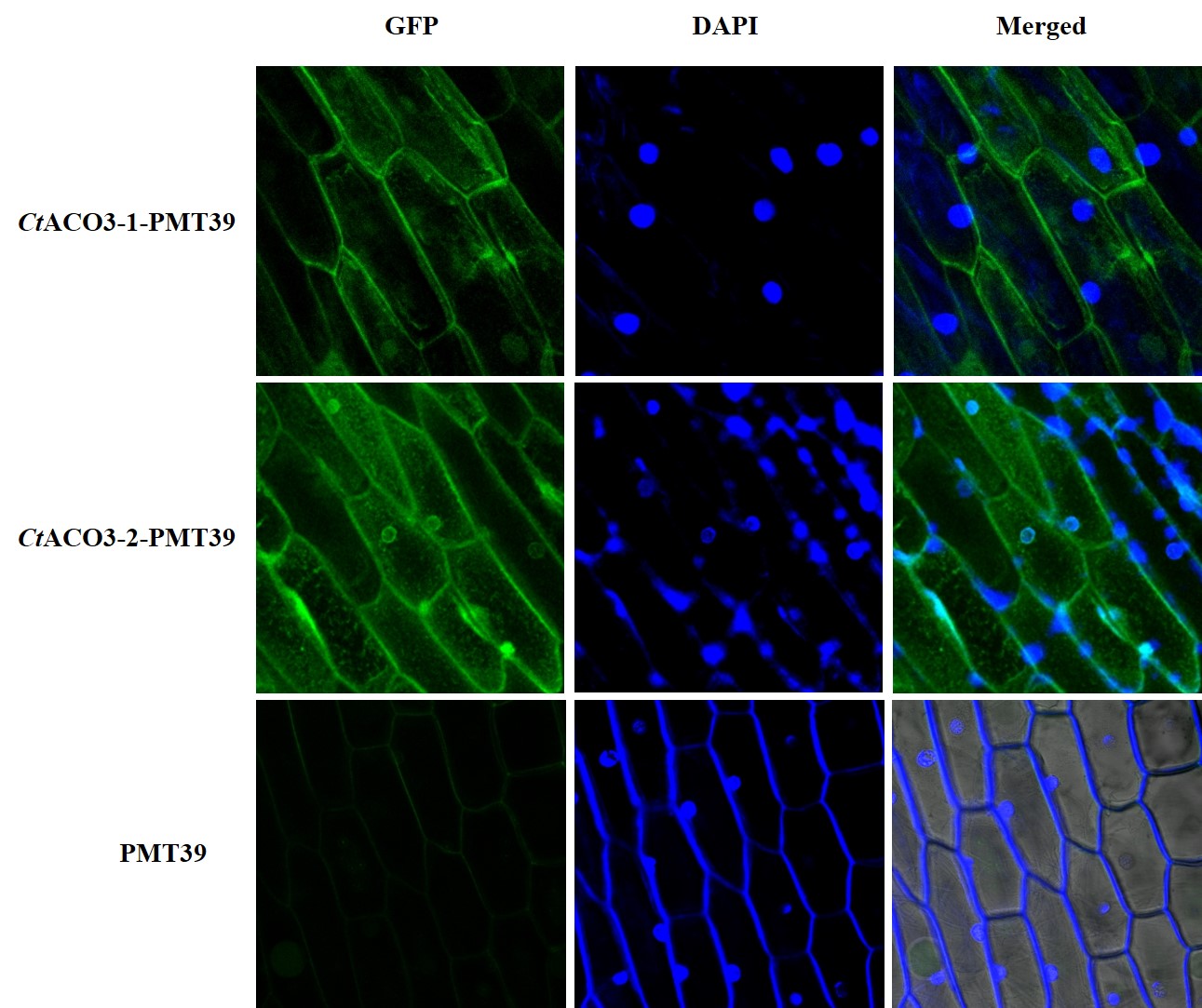

Supplement: Supplementary Figure 2 — Subcellular localization of the CtACO3-PMT39 fusion protein in onion epidermal cells. [file Image_2.JPEG]

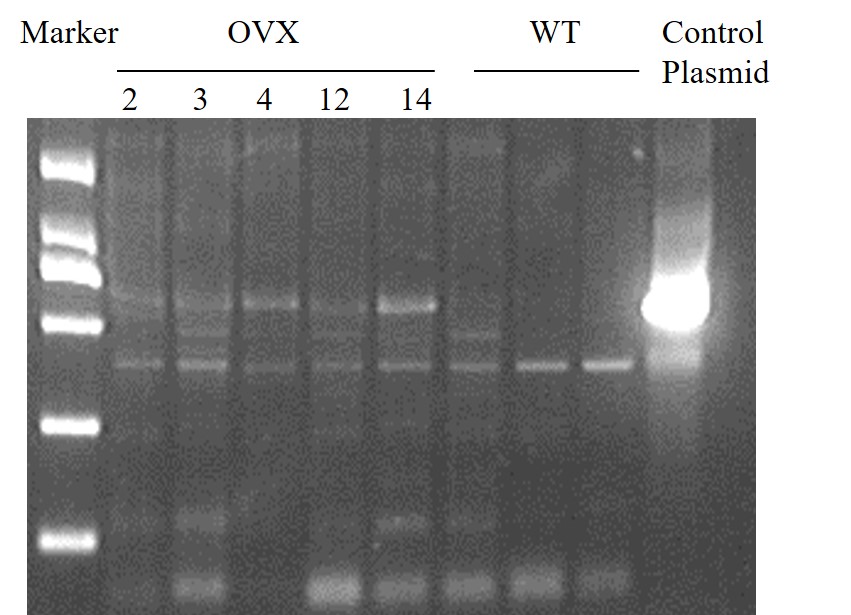

Supplement: Supplementary Figure 3 — Representative PCR analyses for the specific genes of CtACO3-1-overexpressing plants. [file Image_3.JPEG]

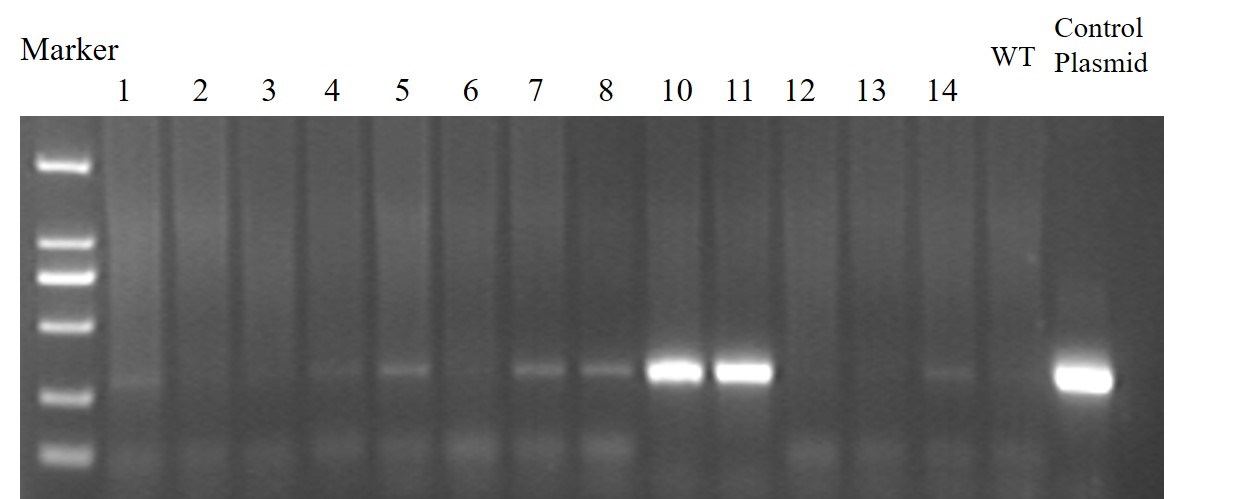

Supplement: Supplementary Figure 4 — Representative PCR analyses for the specific genes of CtACO3-2-overexpressing plants. [file Image_4.JPEG]

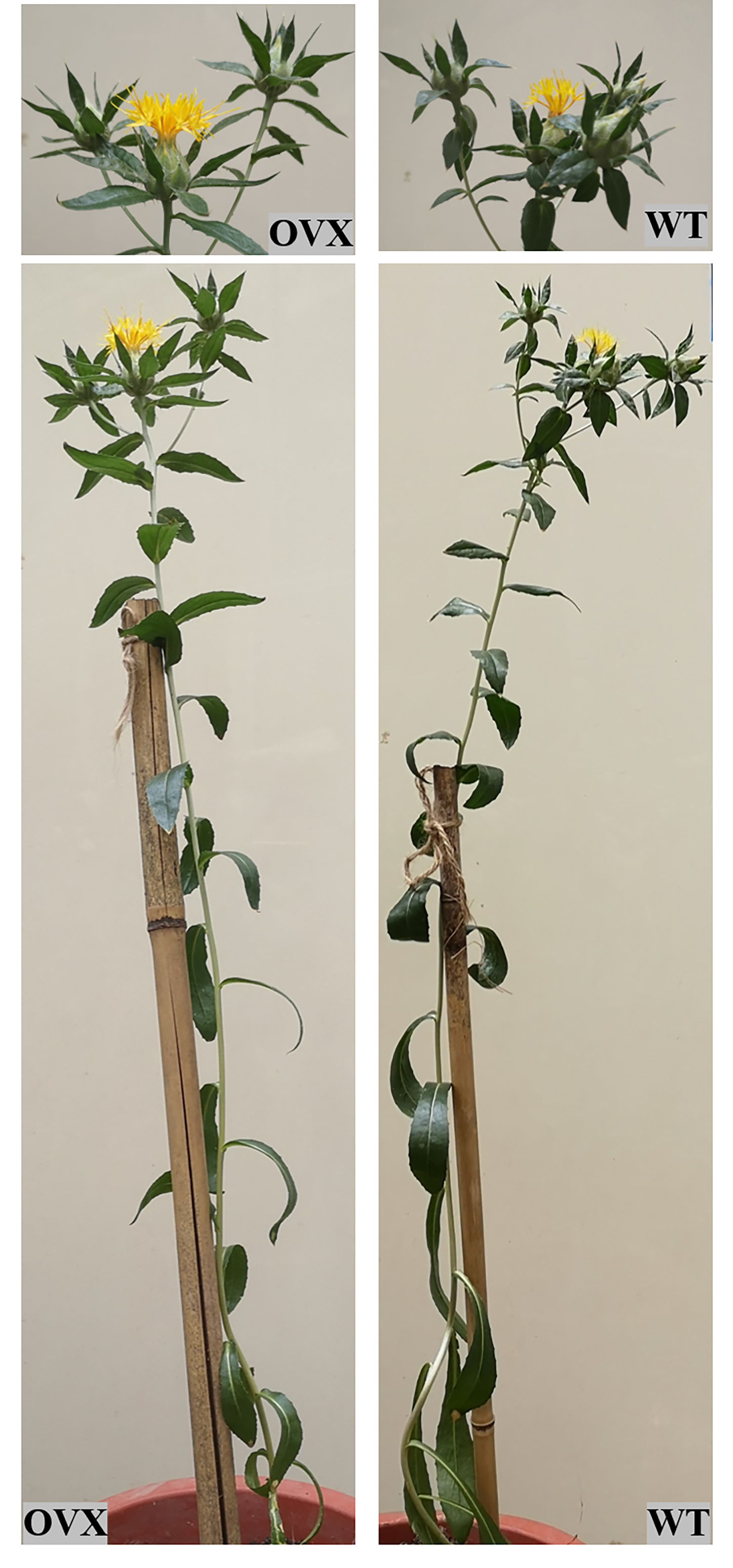

Supplement: Supplementary Figure 5 — Phenotypic difference between WT and CtACO-overexpressing plants. [file Image_5.TIF]

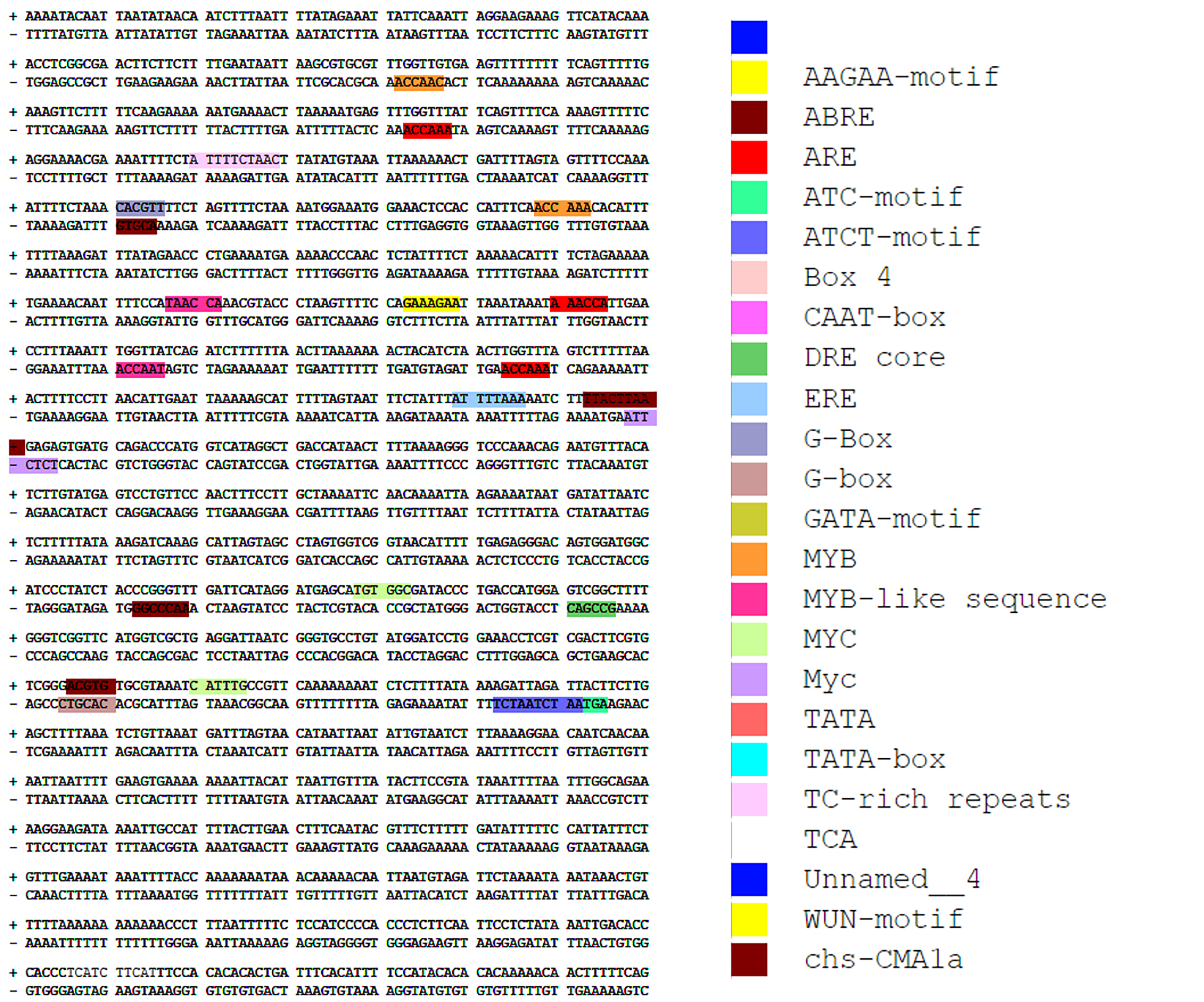

Supplement: Supplementary Figure 6 — Cis-elements in the promoter region of CtACO3-1. [file Image_6.TIF]

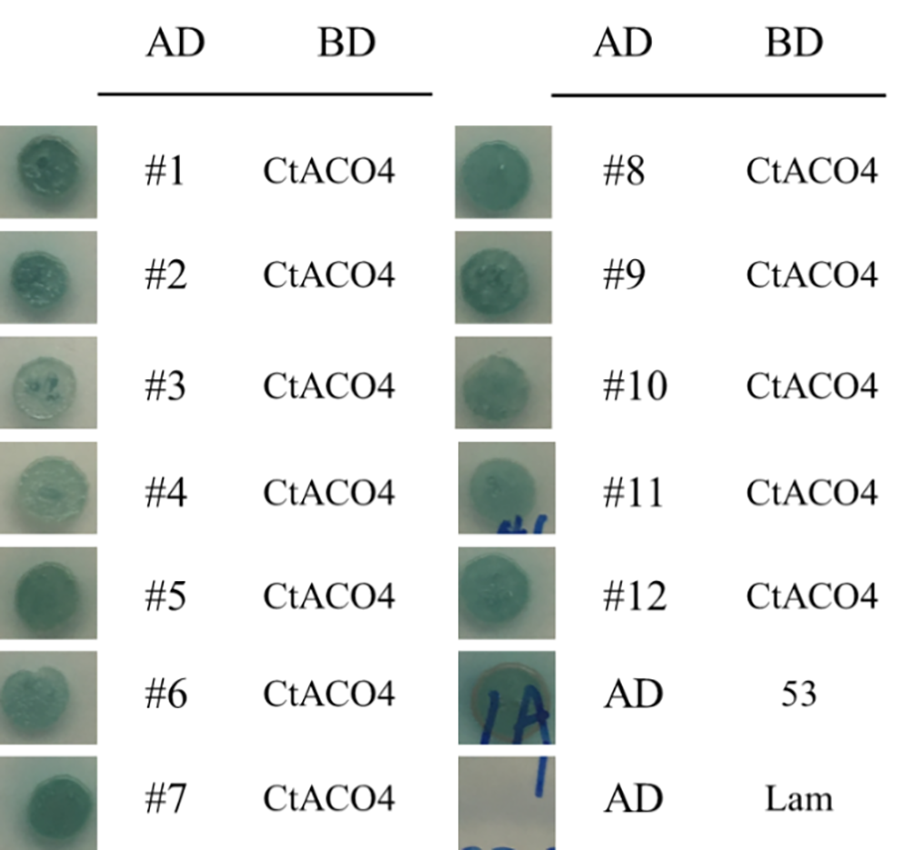

Supplement: Supplementary Figure 7 — Proteins interact with CtACO3-2 by Y2H. [file Image_7.TIF]
