# Supplementary material for: Both Two CtACO3 Transcripts Promoting the Accumulation of the Flavonoid Profiles in Overexpressed Transgenic Safflower
Source: Front Plant Sci. 2022 Apr 6;13:833811. doi: 10.3389/fpls.2022.833811 (PMC9019494; doi:10.3389/fpls.2022.833811)
Supplement: Supplementary Table 1 — PCR primers used in the text. [file Table_1.docx]

| **name** | **sequence** |
| --- | --- |
| ACO3-SP1 | CATACCTCAAAGAATCCCCAGTTC |
| ACO3-SP2 | GGATTTGAACTTGAACCCGTTG |
| ACO3-SP3 | GGTTCAAGCCTATCTCCCACAT |
| CtACO3-1F | ATGGAGGCATTCCCAATTGTGAACAT |
| CtACO3-1R | TTAAACGGTTGCGATCGGGCCAA |
| PMT39-CtACO3-1F | GAGCTTTCGCGGATCCGCCACCATGGGACACACACTGAT |
| PMT39-CtACO3-1R | TCCTCGCCCTTGCTCACCATGGTGGCGGCCGCAACGGTTGCGATC |
| pACO3(-1500~-1000)F | gaaaagcttgaattcgagctcTAAAATACAATTAATATAACAATCTTTAATTTTATAGA |
| pACO3(-1500~-1000)R | agcacatgcctcgaggtcgacATTTAAAGGTTCAATGGTTTATTTATTTAA |
| pACO3(-1000~-500)F | gaaaagcttgaattcgagctcTTGGTTATCAGATCTTTTTTAACTTAAAAA |
| pACO3(-1000~-500)R | agcacatgcctcgaggtcgacATTTACGCACACGTCCCGAC |
| pACO3(-500~-1)F | gaaaagcttgaattcgagctcCATTTGCCGTTCAAAAAAAATCT |
| pACO3(-500~-1)R | agcacatgcctcgaggtcgacTGCTTTCTTAGTTTTCTTCTTTGTTTTT |
| 35SIDF | CGACAGTGGTCCCAAAGAT |
| CtACO3-1IDR1 | CTTCCTGTATTCGTCTTGA |
| CtACO3-2IDR1 | TATGGTCTTCATTGCTTCA |
| CtACO3-2 F | AAAAGTTGGTATCAAGGGTCTGC |
| CtACO3-2 R | ATGGTCTTCATTGCTTCAAACCGAG |
| proGAC | CACGTTCAAAAATATCCACGTTCAAAAATATCCACGTTCAAAAATATCAG |
| proGAC-F | gaaaagcttgaattcgagctcCACGTTCAAAAATATCCACGTTCA |
| proGAC-R | agcacatgcctcgaggtcgacCTGATATTTTTGAACGTGGATATTTTT |
| pGBKT7-ACO3-2 F | aggacctgcatatggccatggATGCACCATTCCATCGTCATC |
| ACO3-2-pGBKT8 R | atgcggccgctgcaggtcgacTTAAACGGTTGCGATCGGG |
| pGBKT7-CNS5a F | aggacctgcatatggccatggATGGTGGTCCATGCCCGC |
| CNS5a-pGBKT7 R | ccgctgcaggtcgacggatccTCAAGATTGAACCATAGGCTCAGG |
| pGADT7- ACO3-2 F | gtaccagattacgctcatatgATGCACCATTCCATCGTCATC |
| ACO3-2-pGADT7 R | cagctcgagctcgatggatccTTAAACGGTTGCGATCGGG |
| 0800-pACO3 F | ctatagggcgaattgggtaccCATTTGCCGTTCAAAAAAAATCT |
| pACO3-0800 R | atctccaccgcggtggcggccgcTGCTTTCTTAGTTTTCTTCTTTGTTTTT |
| SK-ACO3-2 F | ctctctctcaagcttggatccATGCACCATTCCATCGTCATC |
| ACO3-2-SK R | gctcaccatactagtgagctcAACGGTTGCGATCGGGCC |
| ACO3qF | CCGAAGTTTGTGTTTGAG |
| ACO3qR | AACCTTGACCGTATCTATG |
| ACO3-1qF | GATTAGAAGCCGTTCAGA |
| ACO3-1qR | TCGTAGATGTTGGATTCAG |
| PALF | TAATCACCTAAACGGAGTC |
| PALR | CACCATCTTCTTCACCTC |
| C4H1F | TTGTGTAAGGAAGTTAAGGA |
| C4H1R | GTTGTTGTCCATCTTCTTC |
| CHS1F | CACCTTCTGAAAGATGTT |
| CHSIR | GTTCCAATCCGATACC |
| CHI1F | ACTGGTGTTATGGTAGGA |
| CHI1R | GGATACTTCAAGGTAAGGATA |
| F3HF | ACACGAACCGACTATCCATA |
| F3HR | GACCTATCTCCTTCATTCACTT |
| FLSF | CATAGATGCCGTTCTTAATG |
| FLSR | GAGTGGTCTGATGATGAG |
| DFRF | AGGGTGGTAGTGTATTGA |
| DFRR | CATCCAGGCTTTGTCTATA |
| 60SF | CATCCATTATCCAACAATC |
| 60SR | AAGAGTAATCAGTCTCCA |
